# An integrated human behavioral model for mosquito-borne disease control: A scoping review of behavior change theories used to identify key behavioral determinants

**DOI:** 10.1016/j.heliyon.2024.e26488

**Published:** 2024-02-15

**Authors:** Fiona Vande Velde, Hans J. Overgaard, Sheri Bastien

**Affiliations:** a Department of Public Health Science, Faculty of Landscape and Society, Norwegian University of Life Sciences, Ås, Norway; b Department of Translational Physiology, Infectiology and Public Health, Faculty of Veterinary Medicine, Ghent University, Merelbeke, Belgium; c Faculty of Science and Technology, Norwegian University of Life Sciences, Ås, Norway; d Department of Microbiology, Faculty of Medicine, Khon Kaen University, Khon Kaen, Thailand; e Department of Community Health Sciences, Cumming School of Medicine, University of Calgary, Calgary, Canada; f The Centre for Evidence-Based Public Health: A JBI Affiliated Group, Department of Public Health Science, Norwegian University of Life Sciences, Ås, Norway

**Keywords:** Mosquito vector control, Health-protective behaviors, Socio-cognitive theories, Decision-making

## Abstract

Mosquito-borne disease (MBD) control depends largely on a range of public health measures aimed at reducing the spread of infected mosquitoes and human-mosquito contact. These public health measures are generally driven by voluntary, though in few occasions obligatory (e.g., indoor residual spraying), self-protective behaviors by individuals and communities. To develop effective interventions that promote public health measures, the underlying mechanisms that contribute to self-protective behaviors should be well understood. The present scoping review aims to provide a timely overview of how behavior change theories have been applied in the context of MBD control. In addition, the review proposes an integrated model that includes identified key determinants in MBD control behavior, and identifies knowledge gaps to inform future research. A comprehensive search was performed in several databases: MEDLINE, PsycINFO, Embase (Ovid), Web of Science Core Collection, CINAHL, ERIC, and Econ.Lit (EBSCO), as well as registered trials and reviews in CENTRAL and PROSPERO to identify ongoing or unpublished studies. References of included studies and literature reviews were screened, as well as citation tracking in Web of Science, Google Scholar and the malaria database of Behavior Change Impact. This scoping review identified a total of 28 studies. Most studies targeted personal-protective behavioral measures such as adopting, using, or maintaining insecticide-treated bed nets, and were most frequently informed by risk-related behavioral theories. Knowledge and perceived susceptibility of the risk, and related perceived efficacy were identified as key behavioral determinants in the conceptual, integrated human behavior model for MBD control. Numerous studies related to MBD control behavior, especially those focusing on knowledge-attitudes-practices (KAP), often lack a solid theoretical framework, which risks depicting an incomplete understanding of behaviors. In addition, by incorporating various behavioral disciplines into the domain of MBD control, a more comprehensive understanding of key behavioral determinants may be developed and applied in future research and MBD control efforts.

## Highlights

• Studies related to mosquito control behavior often lack a theoretical framework.
• The majority of the included studies target personal-protective measures.
• Often these are informed by risk-related behavioral theories.
• Knowledge and awareness were identified as key behavioral determinants.

## 1 Introduction

Mosquito-borne diseases (MBDs) such as malaria, dengue, Zika, chikungunya, yellow fever, Japanese encephalitis, and West Nile fever, contribute significantly to the global burden of diseases and have a major impact on public health and socio-economic development [1]. More than 80% of the global population is at risk of vector-borne diseases in general, resulting in more than 700,000 annual deaths, of which the vast majority are due to MBDs [1]. MBDs are defined as illnesses caused by pathogens such as viruses, parasites, and bacteria to a lesser extent [2], in human populations carried and transmitted by a mosquito vector. The distribution of these diseases is mostly in tropical and sub-tropical countries and determined by complex dynamics associated with environmental and social factors [3,4]. However, many MBDs are thought to be increasing in incidence and geographic distribution globally, due to climate change and human behaviors [5]. Hence, the increasing importance of effective mosquito vector control.

Mosquito vector control depends largely on a range of public health measures, referred to as non-pharmaceutical interventions (NPIs) aimed at reducing the spread of infected mosquitoes and human-mosquito contact. These interventions include measures that individuals and communities can take to slow the spread of infection especially when vaccines and medical treatments are not available [6]. NPIs can be compulsory measures supported by public health authorities or measures driven by voluntary individual behaviors. The former measures include indoor and outdoor residual spraying (IRS and ORS), space spraying or fogging [7]. The latter, supported by public health campaigns, includes the use of insecticide treated nets (ITN), control of mosquito breeding sites (e.g., eliminating standing water, cleanup campaigns, biological control), or other measures to reduce human-vector contact [7].Voluntary measures have become increasingly important due to documented shortcomings of applying chemical control strategies such as IRS and ITN [8]. However, to be effective, community participation through social mobilization and behavior change is vital to control mosquito breeding sites [4].

Until recently, the status quo in public health interventions targeting behavior change has been to inform and educate communities about the health risks and benefits of different behaviors. A growing body of research now suggests that there is a limited association between knowledge of health benefits and actual performance of a behavior [9], especially when compared to other factors that determine behavior, such as risk perception (i.e., personal evaluation of a specified risk; [10]. A more recent approach is to develop an intervention that targets such key determinants to change behavior [11]. This is done after thoroughly assessing possible factors that positively or negatively influence behaviors in a particular context. Interventions designed by a scientific understanding are often called ‘theory-driven’ since they draw upon a theoretical understanding of how behavior is determined and changed [12]. Interventions developed through theory-driven approaches have been able to demonstrate improvements in the targeted health behavior [13].

To date, there are a total of 83 behavior change theories across the behavioral and social sciences, with as many as 1659 overlapping constructs (i.e., key determinants) [14]. This complex landscape of behavior change theories makes it difficult for researchers, intervention designers, and policymakers to decide which theories to apply and in what context [15]. Therefore, to encourage the use of appropriate behavior change theories and approaches in the design of interventions and MBD control efforts, the aim of the present review is to provide a synthesis of the behavior change theories that have been applied in mosquito vector control strategies, especially focusing on theories to understand and influence individuals' engagement with recommended protective health behaviors. In addition, we aim to map and model the key determinants that have been identified as important drivers of decision-making and behavior in mosquito vector control. In line with these aims and given the diversity of protective health behaviors for vector control, we opted for a scoping review methodology. The specific objectives of this scoping review are to: (1) map existing studies which apply a behavior change theory to measure individuals’ adoption of protective MBD control measures, and provide an overview of the targeted behaviors and behavior change theories utilized, (2) develop a model that includes identified key determinants in MBD control behavior, and (3) identify knowledge gaps to inform future research.

## 2 Methods

A scoping approach was adopted for our search strategy. Scoping reviews are recommended as a mechanism to synthesize a given literature to provide useful insight for decision-makers into the nature of a concept and how it has been studied in the literature over time [16]. Such reviews are particularly useful since they bring together literature from diverse disciplines, and with different approaches to health, intervention development, and measurement outcomes. As the aim of this review was to explore, map and synthesize the literature on behavior and behavior change theories for the adoption of protective MBD control measures, as well as the knowledge gaps, to inform recommendations for public health researchers and decision-makers within MBD control, the adoption of a scoping review framework was an appropriate choice. To ensure a transparent and systematic approach we utilized the JBI Reviewer's Manual methodology for scoping reviews, and the Preferred Reporting Items for Systematic reviews and Meta-Analyses extension for Scoping Reviews (PRISMA-ScR) checklist for reporting [17]. A preliminary search was conducted on March 4, 2021, and no other systematic reviews in the peer-reviewed literature were found that specifically addressed behavior change theories for mosquito-borne disease (MBD) control. In other words, as of that date, the authors did not find any existing systematic reviews in academic journals that were dedicated to exploring behavior change theories in the context of controlling diseases transmitted by mosquitoes.

### 2.1 Search strategy

The search strategy was designed to be as comprehensive and inclusive as possible and combined two main search themes: behavior change theories and MBD control. A systematic search in the following databases was performed: MEDLINE, PsycINFO, and Embase (Ovid); CINAHL, ERIC, and Econ.Lit (EBSCO); Web of Science Core Collection; registered trials and reviews in CENTRAL and PROSPERO to identify relevant in-progress or unpublished studies. Moreover, references of included studies and literature reviews were screened, as well as citation tracking in Web of Science and Google Scholar, and a comprehensive search of the malaria database for Behavior Change Impact (https://behaviorchangeimpact.org/malaria-sbc-evidence-database). The search strategy for all databases, developed and tested in collaboration with a research librarian, is presented in Supplementary file 1.

### 2.2 Selection criteria and screening procedure

The selection criteria were initially developed in conjunction with our search strategy, to be as inclusive as possible. The *a priori* selection criteria simply specified that papers would be included if these contained a behavior change theory pertaining to MBD control practices by a certain population. Only original research articles which included a theoretical underpinning of behavior regarding MBD control were reviewed. Papers were excluded that only used a theoretical underpinning to develop the research instruments without measuring correlations between the determinants as proposed by the theories. Studies that evaluated the effect of a certain behavioral intervention on the determinants or behavior were also excluded. The final inclusion/exclusion criteria are presented in Table 1.

**Table 1:** The eligibility criteria used for this scoping review.

Inclusion criteria	Exclusion criteria
Full-text papers, peer reviewed articles	Conference abstracts, editorial letters and comments, theoretical/background papers, grey literature
Written in English language	Written in other languages than English
Targeted all behavioral practices leading to mosquito vector prevention and control	Targeted other types of behavioral practices leading to prevention and control of other diseases, and behaviors related to case-, or treatment management
Measurement of theoretically informed determinants for understanding behavior	No mention of a behavioral theory/framework
The evaluation of generalizable, baseline determinants and behavior	The evaluation of a behavioral intervention on determinants and behavior
Study design makes it possible to evaluate correlation of the determinant on behavior/self-reported behavior/behavior intention	Study design does not allow for the measuring correlations, such as qualitative studies, literature reviews
Study results report a correlation of the determinant on behavior/self-reported behavior/behavior intention	Analytical methods have not considered measuring the correlations between the determinants

All literature was downloaded by one review author (FVV) to EndNote X9 (Clarivate Analytics, PA, USA), and duplicates removed. The titles of the studies were screened by one reviewer only (FVV) and removed if deemed irrelevant for further inclusion. The remaining studies were imported into Covidence, a web based systematic review platform (Covidence systematic review software, Veritas Health Innovation, Melbourne, Australia). The titles and abstracts were screened independently by two review authors (HJO and FVV). Eligible studies were selected through a questionnaire that specified the inclusion and exclusion criteria. Finally, one review author (FVV) reviewed the full-text studies, whilst a second reviewer (SB) validated 25% of the included studies. Any disagreements concerning eligibility throughout the whole screening process were resolved through discussion.

### 2.3 Data charting

All papers that were selected for inclusion in the review were subjected to a standardized data charting procedure that was developed by the first author and agreed upon by the other review authors. The charting form included the following categories: title; authors; year of publication; journal; country; population; MBD; study design; analytic method; targeted behavior(s); type of protective measure; theory of change; key determinants; determinants with an effect on behavior/self-reported behavior/behavior intention. The charting form was piloted among a sample of five studies comprising different targeted behaviors and using diverse theories of change. The piloting was performed by one review author (FVV) and discussed at length with a second reviewer (SB). Finally, the data charting was performed by one review author (FVV), whilst a second reviewer (SB) validated 25% of the charted information. To map the included studies, the charted data were synthesized in a table, as well as graphically presented to visualize the proposed integrated model.

The diverse study designs and measurements utilized in the included studies varied significantly, making it challenging to evaluate the findings systematically and adequately. Therefore, the aim of the review was to offer a broad perspective of the field rather than conduct a systematic review with appraisal of individual studies. We attempted to charter, summarize and present the findings in an ordinal and visual manner through a conceptual, integrated model, in which the sum scores should *not* be interpreted as factual numbers. To develop the integrated model, data were charted from baseline data collections from the included research articles, i.e. measurements that did not assess the impact of an intervention. Data were synthesized by coding positive effects of behavioral determinants (e.g., social norms) on the behavioral outcome (e.g., adoption of ITN) as “+1” and negative effects as “-1”. Behavioral determinants without a direct effect on the behavioral outcome were not included. Specific beliefs (e.g., the belief that malaria is caused by a mosquito) were not included unless the items were grouped as a behavioral determinant (e.g., barriers to the behaviour). Certain behavioral determinants such as demographics and context were difficult to capture, since these vary in conceptualization and measurement from study to study, and were therefore coded as “varied”. If studies measured multiple separate behaviors, all behavioral determinants affecting each behavior were charted, but only one code per behavioral determinant per study was included. For example, a study that measured two behavioral outcomes (e.g., use of ITN and IRS), which were positively affected by social norms, would receive only “+1” for the behavioral determinant social norms. In case the effect of social norms was both positive and negative, the final code for that study would be 0. Behavioral determinants that received different names but were conceptually similar, based on their description in the studies, were grouped into one model determinant (e.g., perceived severity). Afterward, the number of codes for each model determinant throughout the studies was aggregated in a total sum score. For the visualization of the model, each model determinant was captured by a frame that was linked to the behavioral outcome. The total sum of each model determinant was presented as a green or red arrow, representing a positive or negative effect respectively. The larger total sums were represented through more weighted arrows than the smaller total sums. The model determinants that were difficult to capture due to their variability in conceptualization and measurement were not linked to the behavioral outcome, but rather presented in overall higher-level frames.

## 3 Results

### 3.1 Study selection

**Fig. 1:**
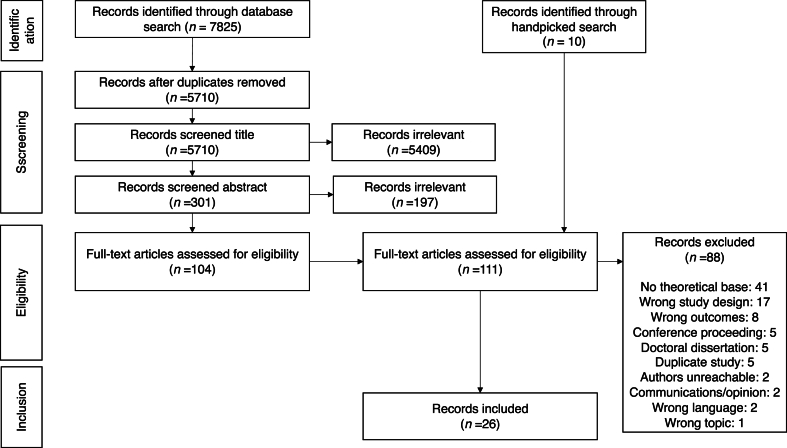
Flow diagram based on the Preferred Reporting Items for Systematic reviews and Meta-Analyses extension for Scoping Reviews (PRISMA-ScR) checklist (Tricco et al., 2018).

The search strategy resulted in a total of 7825 records retrieved from the specified databases on March 12, 2021, with 5710 records remaining after removal of duplicates. The record titles were screened and irrelevant studies removed, and a total of 301 were selected for further screening and imported into Covidence. Another 197 records were removed after screening of the abstracts, resulting in 104 included records for full-text assessment. Following the full-text assessment, 88 records were excluded, whilst 16 records met the inclusion criteria. Another 10 records were included through a handpicked search, which concluded on April 15, 2021. Ultimately, 26 records were identified and charted for review. A PRISMA diagram summarizing the stages of the screening process is presented in Fig. 1. One record included three datasets, collected in different countries and showing different results, and was therefore considered to be three separate studies [18], resulting in a total of 28 studies. The complete list of citations and charted data presented below are available in Supplementary file 2.

### 3.2 Study characteristics

#### 3.2.1 Study locations

There was a wide geographic distribution of studies included. Thirteen of the 28 included studies originated from Africa, including West Africa [19] and ten specified countries: Ethiopia [20,21], Kenya [22], Madagascar [18], Malawi [23], Mali [18], Nigeria [18,24,25], Rwanda [26], Uganda [27], and Tanzania [28]. Ten studies came from the Americas, of which 6 were conducted in the United States [[29], [30], [31], [32], [33], [34]], and one each in Canada [35], Mexico [36], French-Guiana [37], and the Caribbean Island Curacao [38]. Three studies were conducted in Malaysia [[39], [40], [41]], one in the border region of Myanmar with Thailand [42] and another in Australia [43].

#### 3.2.2 Study populations

The main cohort of studies examined community-based populations (16 studies: [19,22,23,[25], [26], [27], [28],^30^,[33], [34], [35], [36],[39], [40], [41], [42]], whilst three studies focused on students in particular [20,37,43]. Another two studies focused on pregnant women, with one examining antenatal care attendees [24], and one examining both pregnant women and women with children under five years old [21]. Three studies which formed one record, focused solely on women with children under five years old [18]. Three studies focused on US travelers, outside of the US in general [31], and in Caribbean destinations in particular [29,32]. One study specifically examined adults with a confirmed chikungunya infection [38].

#### 3.2.3 Mosquito borne diseases

Eleven studies focused on MBDs transmitted by *Aedes* mosquito species, of which four examined dengue fever [36,39,40,43], three chikungunya disease [29,32,38], two Zika fever [30,33], one combining both dengue and chikungunya disease [38], and another combining dengue and Zika [41]. Fourteen studies examined malaria transmitted by *Anopheles* mosquitoes [[18], [19], [20], [21], [22], [23], [24], [25], [26], [27], [28],^42^] Two studies examined West Nile fever transmitted by *Culex* species [34,35]. One study included dengue, Zika, malaria and West Nile fever [31].

### 3.3 Targeted behaviors and theory of change

The types of targeted behaviors in relation to a theory of change in the included studies are presented in Table 2. The MBD control measures were categorized into three types of protective health behaviors: 1) Personal protective behaviors: behaviors enacted by an individual to protect themselves from mosquito bites, e.g. use of mosquito bed nets, clothing or repellents; 2) Mosquito control behaviors: behaviors enacted by an individual, or a group of individuals, to limit mosquito breeding, emergence and presence, e.g. source reduction, window screens, environmental management and manipulation; and 3) Combination behaviors which are a combination of the two previous behaviors (Table 2). The theories of change were sorted into five categories: 1) Risk-related theory: a theory that includes perceptions of risk and its harms and how these relate to behavior; 2) Expectancy-value theory: a theory that includes the evaluation of the expected behavioral outcomes or beliefs; 3) Learning theory: a theory that explains how knowledge and skills are retained and relate to behavior; 4) Context theory: a theory that explains how the context shapes ones behavior and determinants of that behavior; and 5) Combination theory, a combination of different types of theories.

**Table 2:** The types of targeted behaviors in relation to a theory of change in the included studies.

	Behavior	Personal-protective behaviors	Mosquito-control behaviors	Combination behaviors
Theory	Description	Behaviors enacted by an individual to protect themselves from mosquito bites	Behaviors enacted by an individual, or a group of individuals, to limit mosquito breeding, emergence and presence	Combination of personal-protective, and mosquito-control behaviors
Risk-related theory	Explaining how perceptions of risk and its harms and how these relate to behavior	7 [20,21,29,31,32,34,42]	1 [33]	6 [23,30,35,39,41,43]
Expectancy -value theory	Explaining how the evaluation of the expected behavioral outcomes or beliefs relates to behavior	1 [27]	1 [36]	–
Learning theory	Explaining how knowledge and skills are retained and relate to behavior	1 [24]	1 [40]	–
Context theory	Explaining how the context shapes individual behavior and determinants of that behavior	1 [28]	–	–
Combination theory	Combination of different types of theories	6 [18,19,22,25]	1 [38]	2 [26,37]

#### 3.3.1 Targeted behavior

Sixteen studies focused on personal-protective behavioral measures such as adopting, using or maintaining insecticide-treated bed nets [18,[20], [21], [22],^24^,^25^,^27^,^28^,^42^], adopting a certain innovation (Screening and Eave Tubes – SET) [19], using insect repellent [32], or a combination of several personal protective measures (e.g. using repellent, wearing appropriate clothing, avoiding mosquito hours; [29,31,34]. Four studies examined mosquito control behavioral measures, mainly targeting breeding sites and source reduction (e.g. eliminating breeding habitats in standing water and water storage containers, and cleaning of the neighborhood) [36,38,40], and one targeting the mosquito population's receptivity to environmental control (e.g. indoor and outdoor spraying, using larvicide) [33]. The final eight studies measured a combination of different types of protective health measures, from which two combined both personal-protective and mosquito-control measures as one behavioral outcome [23,26,37], another two included both measures in the assessment, but separated the findings [35,43],and three included transmission control measures (e.g. reducing travel, practicing safe sex) in the behavioral outcome mix (39 [30]; 41).

#### 3.3.2 Theory of change

Twelve studies included risk-related theories, of which seven studies applied the Health Belief Model (HBM) [23,31,34,35,39,42,43], two applied the Protection Motivation Theory (PMT) [29,32], one applied a combination of both HBM and PMT [20] and another two applied the Protective Action Decision Model (PADM) [30,33]. Two studies focused on an expectancy-value theory, focusing on the CASCADA Theory of Planned Behavior [36], and Social norms theory [27]. Two studies focused on learning theories, one included the Social Cognitive Theory [40], the other included the Information-Motivation-Behavioral skills model (IMB) [24]. One study focused on a context theory, scrutinizing the role of socio-economic status (SES) [28]. Finally, nine studies examined the role of a combination of theories on protective behaviors: two studies included both context and risk-related theories [26,37]; another study included risk-related and value-expectancy theories [38]; another study combined the diffusion of innovations theory with the integrated model of behavior, both of which can be considered expectancy-value theories to some extent [19]. Five studies examined a conceptual framework that included all different categories of theory: NetWorks conceptual framework [22]; Ideation Model of Strategic Communication and Behavior Change, captured by three studies in one record [18]; and a combinaiton of socio-ecological models of health behaviors, HBM, and Prochaka's stages of change [25].

### 3.4 Integrated human behavior model for MBD control

**Fig. 2:**
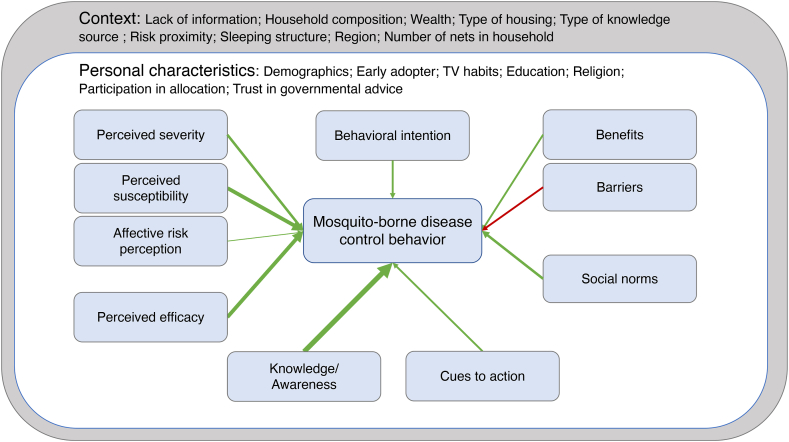
The integrated human behavior model for mosquito-borne disease control, developed by behavioral determinants with an effect on a given targeted behavior in the included studies. Note. Each behavioral determinant is captured by a frame, linked to the behavioral outcome. The total sum of each model determinant is presented as a green or red arrow, representing a positive or negative effect respectively. The larger total sums were represented through more weighted arrows compared to the smaller total sums. (For interpretation of the references to colour in this figure legend, the reader is referred to the Web version of this article.)

The integrated human behavior model for MBD control, visualized in Fig. 2, was developed by combining all behavioral determinants that had an effect on a given target behavior (e.g. self-reported behavior). Table 3 represents all charted behavioral determinants grouped under a model determinant, their labels, and the total sum of the determinants’ scores.

1. *Knowledge/awareness*, the knowledge, the perception about the knowledge, and the awareness of the risk as well as the protective health behaviors, was captured by thirteen studies that presented a positive effect [18,20,22,23,29,31,33,36,37,39,42] and one study presenting a negative effect on the behavioral outcomes [25] resulting in a 12 sum score (13–1).
2. *Perceived susceptibility,* the perception of the susceptibility, vulnerability to the risk and its harm*,* was captured by nine studies [18,29,31,[33], [34], [35],^39^,^42^,^43^], all reporting a positive effect of the variable on the behavioral outcome, resulting in a 9 sum score.
3. *Perceived efficacy*, the perception of the ability and confidence to perform the protective health behavior, captured by ten studies that reported some effect to the variable on the behavioral outcome. Eleven studies reported a positive effect [18,20,23,26,30,32,[36], [37], [38],^40^] while two studies reported a negative effect [22,23], resulting in a 9 sum score (11–2).
4. *Perceived severity*, the perception of the impact of the risk and its harm, was captured by eight studies that reported some effect of the variable on the behavioral outcome. Eight studies reported a positive effect [18,26,29,31,32,41,42], while one study reported a negative effect [22], resulting in a 7 sum score (8–1).
5. *Social norms*, the expectation that others perform or support the protective health behavior, was captured by seven studies that reported some effect on the behavioral outcome. Six studies reported a positive effect [18,20,[25], [26], [27],^36^], while another study reported both a positive and negative effect, depending on the behavioral outcome: intention to adopt an innovation +; intention to maintain an innovation +; intention to diffuse an innovation [19]. The latter study counted two positive effects over one negative and was therefore included as +1, resulting in a sum score of 7 for the model variable.
6. *Benefits,* favorable expected outcomes of the protective health behavior, captured by five studies that reported a positive effect on the behavioral outcome [19,34,36,38,43], resulting in a 5 sum score.
7. *Cue to action*, the stimulus triggering the decision-making process to accept the protective health behavior, was captured by seven studies that reported some effect on the behavioral outcome. Six studies reported a positive effect [24,30,34,35,39], while one study reported a negative effect [19], resulting in a 5 sum score (6–1).
8. *Behavioral intention*, the proximal determinant of behavior that captures the motivation to perform the health protective behavior, captured by four studies, all indicating a positive effect on the behavioral outcome [19,24,26,36], resulting in a 4 sum score.
9. *Affective risk perception*, the affective response to the risk and its harm, captured by one study ([34]) that reported a positive effect on the behavioral outcome, resulting in a 1 sum score.
10. *Barriers,* unfavorable expected outcomes of the protective health behavior, captured by five studies that reported a negative effect on the behavioral outcome [21,25,26,35,39], resulting in a −5 sum score.
11. *Context,* the situation within which the protective health behavior exists or takes place, and helps explain it, captured by five studies, however, all including several variables that had some effect on the behavioral outcome: Lack of information [26]; Household composition and/or wealth [18,23,28] Type of housing and risk proximity [37]; Type of knowledge source and sleeping structure [22] Number of nets in household [18]; Region [18,23]. Since these types of variables are measured differently across studies, or captured by nominal data, the effect cannot be interpreted as an overall positive or negative effect on the behavioral outcome, resulting in a “variable” sum score.
12. *Personal characteristics*, the features or values that belong to an individual and make them recognizable, captured by most studies through the inclusion of demographics, whether as separate variables with an effect on the behavioral outcome or included as a confounder. Another six variables presented some effect on the behavioral outcome: Trust in governmental/advice [30,33]; Early adopter [19]; TV habit [18]; Education [18,23,25]; Religion [18]; Participation in net allocation [18]. Since these types of variables are measured differently across studies, or captured by nominal data, the effect cannot be interpreted as an overall positive or negative effect on the behavioral outcome, resulting in a “variable” sum score.

**Table 3:** The charted behavioral determinants grouped into model determinants, their labels and sum scores included in the integrated human behavioral model for mosquito-borne disease control.

Charted behavioral determinants	Model determinants	Determinant label	Sum score
Knowledge + [20]; Knowledge + [29]; Knowledge + [36]; Knowledge + [39]; Knowledge + [37]; Awareness + (++) [23]; Knowledge beliefs + [23]; Knowledge + [42]; Awareness + [31]; Knowledge + [33]; Malaria knowledge – [25]; Knowledge + [22]; Awareness + [18]; Knowledge to buy + [18]	Knowledge/Awareness	The knowledge, the perception about the knowledge, and the awareness of the risk as well as the protective health behaviors	12
Perceived vulnerability + [29]; perceived susceptibility + [35]; perceived susceptibility + [39]; Susceptibility + [42]; Risk susceptibility + [31]; Perceived personal/community risk + [33]; Perceived threat + [43]; Susceptibility + [34]; Perceived susceptibility + [18]	Perceived susceptibility	The perception of susceptibility, vulnerability to the risk and its harm	9
Self-efficacy + [20]; Control beliefs + [36]; Perceived response/self-efficacy + (++) [26]; Self-efficacy + [38]; Perceived efficacy + [37]; Control beliefs – [23]; Level/strength self-efficacy + [40]; Household/community efficacy + [30]; Perceived response/self- efficacy + [32]; Self-confidence – [22]; Perceived self-efficacy disease + [18]; Response efficacy + [18]; Self-efficacy purchase + [18]	Perceived efficacy	The perception about the ability and confidence to perform the protective health behavior	9
Perceived severity + [29]; Perceived severity + [26]; Seriousness + [42]; Perceived severity + [32]; Risk severity + [31]; Perception of risk - [22]; Perceived threat + [43]; Perceived severity + [41]; Perceived severity + [18]	Perceived severity	The perception about the impact of the risk and its harm	7
Family support + [20]; Social norms + [36]; Subjective norms + [26]; Perceived social norm + [27]; Social support + [25]; Social norms + (+/+/−) [19]; Perceived norm + [18]	Social norms	The perception about the expectation of others in performing the protective health behavior	7
Attitudes + [36]; Attitudes + [38]; Attitudes + [19]; Cost effectiveness + [43]; Benefit + [34]	Benefits	Favorable expected outcomes of the protective health behavior	5
Cue to action + [35]; Information + [24]; Cue to action + [39]; Information + [30]; WOM received – [19]; Cues + [34]; Message exposure + [18]	Cue to action	The stimulus triggering the decision-making process to accept the protective health behavior	5
Intention to change + [36]; Behavioral intentions + [26]; Motivation + [24]; WOM shared + [19];	Behavioral intention	The proximal determinant of behavior that captures the motivation to perform the health protective behavior	4
Affective risk perception + [34]	Affective risk perception	The affective response to the risk and its harm	1
Perceived barriers – [35]; Perceived discomfort – [26]; perceived barriers – [39]; Reported disadvantages – [25]; Perceived barriers – [21]	Barriers	Unfavorable expected outcomes of the protective health behavior	−5
Lack of information [26]; Household composition [18,28]; Wealth/SES [18,23,28]; Type of housing [37]; Risk proximity [37]; Local control implemented [23]; Type of knowledge source [22]; Sleeping structure [22]; Nets in household [18]; Region/Vilage [18,23]	Context	The situation within which the protective health exists or happens, and helps explain it	Varied
Education [18,23,25]; Trust in governmental/advice [30,33]; Early adopter [19]; TV-habits [18]; Religion [18]; Participation in allocation [18]	Personal characteristics	The features or values that belong to a person and make them recognizable	Varied

## 4 Discussion

This scoping review incorporated 26 articles, encompassing a total of 28 studies that examined the application of behavior change theories in the context of MBD control. The review offers three primary contributions: Firstly, it provides a comprehensive overview of the behaviors targeted and the behavior change theories utilized to measure individuals’ adoption of protective MBD control. The majority of studies focused on personal-protective behaviors such as adopting, using, or maintaining insecticide-treated bed nets. Risk-related behavioral theories were frequently employed in these studies. Secondly, the review presents a conceptual, integrated human behavior model that identifies key determinants of MBD control behavior. The analysis revealed that knowledge and perceived susceptibility of the risk, and related perceived efficacy were identified as crucial factors. Thirdly, the review identifies knowledge gaps to inform future research. It highlights a lack of solid theoretical frameworks in numerous studies related to MBD control behavior, particularly those focusing on knowledge-attitudes-practices (KAP). This deficiency risks an incomplete understanding of behaviors. To address this, the incorporation of diverse behavioral disciplines into the domain of MBD control is recommended, enabling a more comprehensive understanding of key determinants of behavior in future research and MBD control efforts. Overall, this synthesis offers valuable recommendations and suggestions for researchers and public health professionals seeking to apply behavior change theory in their understanding and influence of MBD behavior.

The landscape of behavioral theory is highly complex and confusing, and therefore difficult to navigate. To improve the uptake of theory in the context of MBDs, this review mapped the application of theories of change for MBD control behavior. Firstly, it aimed to understand the protective health behaviors targeted for MBD control, since behavior is central to choosing an appropriate theory. The more specific a certain behavior is identified and described, the closer its measurement will relate to the real-life situation, since different behavioral actions are influenced by different determinants. The majority of the studies included in the review showed adequate specificity in relation to the targeted behaviors (e.g. adoption of insecticide treated bed nets, use of insect repellents). However, some studies combined different actions into one behavioral outcome, such as personal-protective behaviors [22,31,34]. This is acceptable if the actions are not vastly different from one another, which can result, primarily, in an unsuitable behavioral measurement scale, and secondly, results that fail to adequately reflect the real-life situation. One such example is presented by Smith and colleagues [19], where social norms had opposite effects on different behavioral actions, even though all actions related to personal-protective behaviors. For this purpose, the review categorized the targeted behaviors into personal-protective and mosquito-control behaviors, each representing similar protective health measures. A final category, combination of behaviors, was developed out of necessity since several studies combined distinct protective health measures into one behavioral outcome. This type of research risks decreasing the validity of the study results if adequate and appropriate statistical analyses are not performed. Structural equation modeling is a group of methods, recommended for measuring such models since it generally involves both factor analysis and path analysis [44], and presents a comprehensive model of behavior.

Secondly, this review aimed to explore possible patterns of targeted behavior with a specified theory of change. Apart from risk-related theories being implemented most frequently for personal-protective and combination behaviors, no other possible patterns were deduced. Selecting risk-related theories such as the Health Belief Model, the Protection Motivation Theory, and the Protective Action Decision Model for the evaluation and prediction of health behaviors is in line with research across other health behaviors [15]. It makes sense that studies focusing on personal-protective behaviors, also included in combination behaviors, would select a risk-related theory, since the risk is more salient with regards to these behaviors. The behaviors focus explicitly on the protection against mosquito bites, which are directly linked to health risk, that is, the potential transmission of a mosquito-borne pathogen. Mosquito-control behaviors, on the other hand, are more adopted to reduce general mosquito abundance than with direct risk reduction of transmission. Applying a context theory to mosquito control behaviors would be more advantageous because these actions typically target changing the environment, and hence the context. Therefore, we recommend specifying the protective health behavior, or at least the category of behaviors, as the basis for selecting an appropriate theory underpinned by the characteristics of the behavior. Another suitable approach would be to select a framework that includes different categories of theory, such as NetWorks (22) and Ideation [18]. This would allow for comparison across different study designs (e.g., targeted behaviors, populations, regions), and eventually result in a more comprehensive understanding of MBD control behaviors.

Finally, the integrated human model for MBD control was developed by synthesizing the outcomes from the included studies in a purposeful way, such that it includes all determinants with an effect on the behavioral outcomes. Knowledge and awareness of the risk received the largest total sum and were therefore identified as the main behavioral determinants of MBD control. This contradicts the growing body of research that shows a limited association between knowledge of health benefits and actual performance of a behavior [9], but stresses the importance of health education and information. Perceived susceptibility and perceived severity of the risk, not surprisingly, were assessed repeatedly through risk-related models, and identified as important determinants of MBD control behaviors. This finding is valuable for designing effective public health interventions. However, further research would greatly benefit from exploring the characteristics and dynamics of these key determinants to gain a deeper understanding of their impact. A study by Raude and colleagues [45] explored the dynamic interaction between risk-related perceptions and behaviors that occur in response to a large chikungunya epidemic. They found that risk perception of contracting the disease decreased during the course of the outbreak, which may be attributed to risk habituation effects. Risk habituation refers to a behavioral phenomenon where individuals progressively underestimate or disregard risks as they become more familiar with a particular health threat. The extent of this pattern appears to vary significantly depending on the type of intervention used, whether it involves personal-protective approaches or environmental control strategies. This emphasizes the importance of distinguishing between different behavior types and selecting appropriate theories when studying risk habituation. Additionally, it highlights the necessity of incorporating various behavioral disciplines, such as behavioral economics, into the field of MBD control. By doing so, a more comprehensive understanding of the behaviors underlying MBD control can be developed which may lead to the design of more effective public health interventions that are specifically tailored to the characteristics of different populations and behaviors.

Since the aim of the review was to provide an overview of the field and not appraise studies as in a systematic review, the results from this section – the sum scores – should *not* be interpreted as factual numbers, but rather as an attempt to condense and represent the findings in an ordinal and visual way. Moreover, the study designs and measurements differ greatly amongst the included studies, which makes it difficult to evaluate the findings systematically and adequately. Nevertheless, the illustrated model (Fig. 2) consolidates the review findings into a comprehensive, accessible manner, which can be the basis for future research. Future research could potentially validate the model, or build on it by including aspects from other types of research such as qualitative studies and participatory workshops with experts, as was the case for the integrated behavioral model for water, sanitation and hygiene (IBM-WASH) [10].

Based on the assessment of eligibility, many studies, knowledge-attitude-practice (KAP) studies in particular, were excluded from the analysis due to the lack of a specified theoretical foundation. These studies were similar to the studies included in this review, but were generally baseline measurements to inform future public health interventions [46,47]. If a study failed to specify its theoretical foundation and didn't have a baseline measurement, it was considered to be lacking in providing clear and organized information about the best approaches to change behavior, as explained by behavior change theories [48]. Without the systematic application of a theory of change, a study risks missing out on a comprehensive perspective of behavior. Although providing meaningful insights, the results of such studies are considered less useful for underpinning a public health intervention [49]. Therefore, a notable recommendation of this review is directed toward researchers that plan to undertake similar studies. Future studies in this area should include a clear specification of the theory of change used to develop an intervention.

### 4.1 Limitations of the review

#### 4.1.1 Search strategy

The objective of the search strategy was to provide a comprehensive overview of the behaviors targeted and the behavior change theories utilized to measure individuals' adoption of protective MBD control, without limiting the review to studies that solely rely on socio-cognitive theories. However, it's worth noting that the majority of the search terms used were primarily derived from socio-psychological research, which might have led to overlooking behavioral research that is theoretically grounded in other areas (e.g., behavioral economics).

#### 4.1.2 Selection criteria

The selection criteria were designed to exclude studies that did not clearly state an underlying behavior theory or framework. It is important to note that different disciplines, such as behavioral economics, may use different terminology, ways of citing or labeling theory. Without this specific background information, many relevant studies could have been left out of this review. Additionally, this review only considered peer-reviewed articles to ensure a more focused and scientifically rigorous assessment. However, it is worth acknowledging that there may be studies of scientific rigor to be found in other sources, such as grey literature, that were excluded based on this criteria. Ultimately, the review's scope is confined to works exclusively written in English, potentially leading to the omission of information presented in other languages. This limitation is particularly noteworthy in the context of South America, a continent grappling with numerous mosquito-borne diseases. Research in this region is predominantly conveyed and disseminated in Spanish and Portuguese. The exclusion of non-English literature may result in the loss of valuable insights.

#### 4.1.3 Data charting

The review did not specify the type of behavioral outcomes (e.g., behavioral intention, self-reported behavior, observed behavior) and merged all outcomes into one final category. However, some studies included behavioral intention as a determinant of the final outcome, while others had behavioral intention as the final outcome. The review therefore acknowledges some bias, and missed opportunity, in reporting the final behavioral outcome.

## Data availability statement

The authors confirm that the data supporting the findings of this study are available within the article and its supplementary materials.

## CRediT authorship contribution statement

**Fiona Vande Velde:** Writing – review & editing, Writing – original draft, Visualization, Investigation, Formal analysis, Conceptualization. **Hans J. Overgaard:** Writing – review & editing, Writing – original draft, Visualization, Supervision, Investigation, Funding acquisition, Conceptualization. **Sheri Bastien:** Writing – review & editing, Writing – original draft, Visualization, Supervision, Investigation, Conceptualization.

## Declaration of generative AI and AI-assisted technologies in the writing process

During the preparation of this work, the authors used QuillBot (Course Hero, LLC, 2023) in order to improve language and readability. After using this tool/service, the authors reviewed and edited the content as needed and take(s) full responsibility for the content of the publication.

## Declaration of competing interest

The authors declare the following financial interests/personal relationships which may be considered as potential competing interests:Hans J. Overgaard reports financial support was provided by 10.13039/501100005416Research Council of Norway. If there are other authors, they declare that they have no known competing financial interests or personal relationships that could have appeared to influence the work reported in this paper.
