# Supplementary material for: An integrated human behavioral model for mosquito-borne disease control: A scoping review of behavior change theories used to identify key behavioral determinants
Source: Heliyon. 2024 Feb 15;10(4):e26488. doi: 10.1016/j.heliyon.2024.e26488 (PMC10901007; doi:10.1016/j.heliyon.2024.e26488)
Supplement: Multimedia component 1 [file mmc1.docx]

*Search Strategy Ovid*

| Embase | Medline | PsychINFO | Free keywords |
| --- | --- | --- | --- |
| Exp behavior theory/ or psychological model/ or cognitive model/ or conceptual framework/ or theoretical model/ or change theory/ or decision theory/ or theory validation | Exp models, psychological/ or models, Theoretical/ or attitude/ or attitude to health/ or health behavior/ or Psychology, Social/ or Exp Health belief model/ | Reasoned Action/ or Exp Planned Behavior/ or Exp Behavior Change/ or Health Belief Model/ or Exp Transtheoretical Model/ or Exp Decision Making/ or Exp Decision Theory/ or Intention/ or Exp Motivation/ or Exp Attitudes/ or Exp Risk perception/ or Exp Social norms/ | **Unbalanced index indicators**  Øverst i skjemaet  ((behavioral or motivation or *cognitive or *psycho* or *socio* or stage* or action or goal or organization* or *ecological or (decision making)) adj (model* or framework* or theor*)).tw,kf. |
| ((behavio?ral or motivation or cognitive or psycho* or socio* or stage* or action* or goal* or organization* or ecological or "decision making") adj2 (model* or framework* or theor*)).tw,kw. | ((behavio?ral or motivation or cognitive or psycho* or socio* or stage* or action* or goal* or organization* or ecological or "decision making") adj2 (model* or framework* or theor*)).tw,kf. | ((behavio?ral or motivation or cognitive or psycho* or socio* or stage* or action* or goal* or organization* or ecological or "decision making") adj2 (model* or framework* or theor*)).tw. | ((behavio?ral or motivation or cognitive or psycho* or socio* or stage* or action* or goal* or organization* or ecological or "decision making") adj2 (model* or framework* or theor*)) |
|  |  |  | ((theory adj2 (planned behavio?r) or (reasoned action)) or (stages of change) or (diffusion of innovations) or (COM-B)).tw,kf. |
| ((theory adj2 ("planned behavio?r" or "reasoned action*")) or "stages of change" or "diffusion of innovation*" or COM-B).tw,kw. | ((theory adj2 ("planned behavio?r" or "reasoned action*")) or "stages of change" or "diffusion of innovation*" or COM-B).tw,kf. | ((theory adj2 ("planned behavio?r" or "reasoned action*")) or "stages of change" or "diffusion of innovation*" or COM-B).tw. |  |
|  |  |  | ((behavioral game) or (protection motivation) or (health belief) or (social cognitive) or (locus of control) or (social learning) or (social comparison) or (cognitive adaptation) or (social identity) or (elaboration likelihood) or self-determination or attribution or (social judgment) or (dual processing) or learning or operant or self-regulation or automotive or (volitional control) or (social cognitive) or (cognitive behavior) or (transtheoretical) or (social identity) or effort-reward or demand-control or diffusion or (group minority) or (person situation contingency) or (value expectancy) or (capability opportunity motivation) or (social regulation) or (antecedent behavior consequence) or (ABC)  Adj1 (model* or framework* or theor*)).tw,kf. |
| ("behavio?ral game*" or "protection motivation" or "health belie*" or "social cognitive" or "locus of control" or "social learning" or "social comparison" or "cognitive adaptation*" or "social identit*" or "elaboration likelihood" or "self-determination" or attribution* or "social judgment*" or "dual processing" or learning or operant or "self-regulation" or automotive or "volitional control" or "social cognitive" or "cognitive behavior" or transtheoretical or "effort-reward" or "demand-control" or diffusion or "group minorit*" or "person situation contingency" or "value expectanc*" or "capability opportunity motivation" or "social regulation*" or "antecedent behavior consequence*" or ABC).tw,kw. | ("behavio?ral game*" or "protection motivation" or "health belie*" or "social cognitive" or "locus of control" or "social learning" or "social comparison" or "cognitive adaptation*" or "social identit*" or "elaboration likelihood" or "self-determination" or attribution* or "social judgment*" or "dual processing" or learning or operant or "self-regulation" or automotive or "volitional control" or "social cognitive" or "cognitive behavior" or transtheoretical or "effort-reward" or "demand-control" or diffusion or "group minorit*" or "person situation contingency" or "value expectanc*" or "capability opportunity motivation" or "social regulation*" or "antecedent behavior consequence*" or ABC).tw,kf. | ("behavio?ral game*" or "protection motivation" or "health belie*" or "social cognitive" or "locus of control" or "social learning" or "social comparison" or "cognitive adaptation*" or "social identit*" or "elaboration likelihood" or "self-determination" or attribution* or "social judgment*" or "dual processing" or learning or operant or "self-regulation" or automotive or "volitional control" or "social cognitive" or "cognitive behavior" or transtheoretical or "effort-reward" or "demand-control" or diffusion or "group minorit*" or "person situation contingency" or "value expectanc*" or "capability opportunity motivation" or "social regulation*" or "antecedent behavior consequence*" or ABC).tw. |  |
| Exp behavioral economics/ | exp Economics, Behavioral/ | Exp Behavioral Economics/ | ((behavio?ral adj2 economic*) or (bound* adj1 rational*) or (cognitive adj1 (effect* or bias*)) or heuristic* or fallac*).tw,kf. |
| ((behavio?ral adj2 economic*) or (bound* adj2 rational*) or (cognitive adj2 (effect* or bias*)) or heuristic* or fallac*).tw,tw. | ((behavio?ral adj2 economic*) or (bound* adj2 rational*) or (cognitive adj2 (effect* or bias*)) or heuristic* or fallac*).tw,kf | ((behavio?ral adj2 economic*) or (bound* adj2 rational*) or (cognitive adj2 (effect* or bias*)) or heuristic* or fallac*).tw. |  |
|  |  |  | AND |
| Exp Insect Control/ or Exp bed net/ or Exp insect repellent/ or Exp insect vector | exp Insect Control/ or insecticides/ or Insect Repellents/ or Exp Mosquito Nets/ or Exp Insect Vectors/ or Fumigation/ | Exp Insecticides/ | (((Vector or mosquito* or insect* or tick*) adj (control or management)) or IVM or repellent* or insecticide or ovitrap* or (BG sentinel trap*) or (insecticide treated material*) or (bed net*) or LLIN or (long lasting insecticidal net*) or (insecticide treated net*) or (ITN) or larvicide or ((larv* or guppy) adj1 fish) or ((remov* or clear* or dispos* or eliminat*) adj2 (breed* or source*)) or (cover* adj2 (container* or lid*))) |
| (((Vector or mosquito* or insect* or tick*) adj2 (control or management)) or IVM or repellent* or insecticide or ovitrap* or "BG sentinel trap*" or "insecticide treated material*" or "bed net*" or LLIN or "long lasting insecticidal net*" or "insecticide treated net*" or ITN or larvicide or ((larv* or guppy) adj2 fish) or ((remov* or clear* or dispos* or eliminat*) adj2 (breed* or source*)) or (cover* adj2 (container* or lid*))).tw,kw. | (((Vector or mosquito* or insect* or tick*) adj2 (control or management)) or IVM or repellent* or insecticide or ovitrap* or "BG sentinel trap*" or "insecticide treated material*" or "bed net*" or LLIN or "long lasting insecticidal net*" or "insecticide treated net*" or ITN or larvicide or ((larv* or guppy) adj2 fish) or ((remov* or clear* or dispos* or eliminat*) adj2 (breed* or source*)) or (cover* adj2 (container* or lid*))).tw,kf. | (((Vector or mosquito* or insect* or tick*) adj2 (control or management)) or IVM or repellent* or insecticide or ovitrap* or "BG sentinel trap*" or "insecticide treated material*" or "bed net*" or LLIN or "long lasting insecticidal net*" or "insecticide treated net*" or ITN or larvicide or ((larv* or guppy) adj2 fish) or ((remov* or clear* or dispos* or eliminat*) adj2 (breed* or source*)) or (cover* adj2 (container* or lid*))).tw. |  |

*Search Strategy Ebsco*

| Cinhal | ERIC | ECON.lit | Free keywords |
| --- | --- | --- | --- |
| (MH "Conceptual Framework") OR (MH "Change Theory") OR (MH "Grounded Theory") OR (MH "Models, Theoretical") OR (MH "Health Belief Model") OR (MH "Models, Psychological+") OR (MH "Psychological Theory+") OR (MH "Theory Construction") OR (MH "Theory Validation") OR (MH "Control (Psychology)+") | (DE "Conceptual Framework") OR (DE "Change Theory") OR (DE "Grounded Theory") OR (DE"Models, Theoretical") OR (DE "Health Belief Model") OR (DE "Models, Psychological+") OR (DE "Psychological Theory+") OR (DE "Theory Construction") OR (DE "Theory Validation") OR (DE "Control (Psychology)+") |  | **Unbalanced index indicators**  Øverst i skjemaet  ((behavioral or motivation or *cognitive or *psycho* or *socio* or stage* or action or goal or organization* or *ecological or (decision making)) W0 (model* or framework* or theor*)).tw,kf. |
| (behavio#ral or motivation or cognitive or psycho* or socio* or stage* or action* or goal* or organization* or ecological or "decision making") N1 (model* or framework* or theor*) | (behavio#ral or motivation or cognitive or psycho* or socio* or stage* or action* or goal* or organization* or ecological or "decision making") N1 (model* or framework* or theor*) | (behavio#ral or motivation or cognitive or psycho* or socio* or stage* or action* or goal* or organization* or ecological or "decision making") N1 (model* or framework* or theor*) | ((behavio#ral or motivation or cognitive or psycho* or socio* or stage* or action* or goal* or organization* or ecological or "decision making") N1 (model* or framework* or theor*)) |
|  |  |  | ((theory N1 (planned behavio#r) or (reasoned action)) or (stages of change) or (diffusion of innovations) or (COM-B)).tw,kf. |
| (theory N1 ("planned behavio#r" or "reasoned action*")) or "stages of change" or "diffusion of innovation*" or COM-B | (theory N1 ("planned behavio#r" or "reasoned action*")) or "stages of change" or "diffusion of innovation*" or COM-B | (theory N1 ("planned behavio#r" or "reasoned action*")) or "stages of change" or "diffusion of innovation*" or COM-B |  |
|  |  |  | ((behavioral game) or (protection motivation) or (health belief) or (social cognitive) or (locus of control) or (social learning) or (social comparison) or (cognitive adaptation) or (social identity) or (elaboration likelihood) or self-determination or attribution or (social judgment) or (dual processing) or learning or operant or self-regulation or automotive or (volitional control) or (social cognitive) or (cognitive behavior) or (transtheoretical) or (social identity) or effort-reward or demand-control or diffusion or (group minority) or (person situation contingency) or (value expectancy) or (capability opportunity motivation) or (social regulation) or (antecedent behavior consequence) or (ABC)  W0 (model* or framework* or theor*)).tw,kf. |
| "behavio#ral game*" or "protection motivation" or "health belie*" or "social cognitive" or "locus of control" or "social learning" or "social comparison" or "cognitive adaptation*" or "social identit*" or "elaboration likelihood" or "self-determination" or attribution* or "social judgment*" or "dual processing" or learning or operant or "self-regulation" or automotive or "volitional control" or "social cognitive" or "cognitive behavior" or transtheoretical or "effort-reward" or "demand-control" or diffusion or "group minorit*" or "person situation contingency" or "value expectanc*" or "capability opportunity motivation" or "social regulation*" or "antecedent behavior consequence*" or ABC | "behavio#ral game*" or "protection motivation" or "health belie*" or "social cognitive" or "locus of control" or "social learning" or "social comparison" or "cognitive adaptation*" or "social identit*" or "elaboration likelihood" or "self-determination" or attribution* or "social judgment*" or "dual processing" or learning or operant or "self-regulation" or automotive or "volitional control" or "social cognitive" or "cognitive behavior" or transtheoretical or "effort-reward" or "demand-control" or diffusion or "group minorit*" or "person situation contingency" or "value expectanc*" or "capability opportunity motivation" or "social regulation*" or "antecedent behavior consequence*" or ABC | "behavio#ral game*" or "protection motivation" or "health belie*" or "social cognitive" or "locus of control" or "social learning" or "social comparison" or "cognitive adaptation*" or "social identit*" or "elaboration likelihood" or "self-determination" or attribution* or "social judgment*" or "dual processing" or learning or operant or "self-regulation" or automotive or "volitional control" or "social cognitive" or "cognitive behavior" or transtheoretical or "effort-reward" or "demand-control" or diffusion or "group minorit*" or "person situation contingency" or "value expectanc*" or "capability opportunity motivation" or "social regulation*" or "antecedent behavior consequence*" or ABC |  |
|  |  |  | ((behavio#ral N1 economic*) or (bound* N1 rational*) or (cognitive N1 (effect* or bias*)) or heuristic* or fallac*).tw,kf. |
| (behavio#ral N1 economic*) or (bound* N1 rational*) or (cognitive N1 (effect* or bias*)) or heuristic* or fallac* | (behavio#ral N1 economic*) or (bound* N1 rational*) or (cognitive N1 (effect* or bias*)) or heuristic* or fallac* | (behavio#ral N1 economic*) or (bound* N1 rational*) or (cognitive N1 (effect* or bias*)) or heuristic* or fallac* |  |
|  |  |  | AND |
| (MH "Insect Repellents") OR (MH "Pest Control+") OR (MH "Mosquito Nets") | (DE "Insect Repellents") OR (DE "Pest Control+") OR (DE "Mosquito Nets") |  | (((Vector or mosquito* or insect* or tick*) W0 (control or management)) or IVM or repellent* or insecticide or ovitrap* or (BG sentinel trap*) or (insecticide treated material*) or (bed net*) or LLIN or (long lasting insecticidal net*) or (insecticide treated net*) or (ITN) or larvicide or ((larv* or guppy) N0 fish) or ((remov* or clear* or dispos* or eliminat*) N1 (breed* or source*)) or (cover* N1 (container* or lid*))) |
| ((Vector or mosquito* or insect* or tick*) N1 (control or management)) or IVM or repellent* or insecticide or ovitrap* or "BG sentinel trap*" or "insecticide treated material*" or "bed net*" or LLIN or "long lasting insecticidal net*" or "insecticide treated net*" or ITN or larvicide or ((larv* or guppy) N1 fish) or ((remov* or clear* or dispos* or eliminat*) N1 (breed* or source*)) or (cover* N1 (container* or lid*)) | ((Vector or mosquito* or insect* or tick*) N1 (control or management)) or IVM or repellent* or insecticide or ovitrap* or "BG sentinel trap*" or "insecticide treated material*" or "bed net*" or LLIN or "long lasting insecticidal net*" or "insecticide treated net*" or ITN or larvicide or ((larv* or guppy) N1 fish) or ((remov* or clear* or dispos* or eliminat*) N1 (breed* or source*)) or (cover* N1 (container* or lid*)) | ((Vector or mosquito* or insect* or tick*) N1 (control or management)) or IVM or repellent* or insecticide or ovitrap* or "BG sentinel trap*" or "insecticide treated material*" or "bed net*" or LLIN or "long lasting insecticidal net*" or "insecticide treated net*" or ITN or larvicide or ((larv* or guppy) N1 fish) or ((remov* or clear* or dispos* or eliminat*) N1 (breed* or source*)) or (cover* N1 (container* or lid*)) |  |

*Search Strategy WoS*

| WoS | Free keywords |
| --- | --- |
|  | **Unbalanced index indicators**  Øverst i skjemaet  ((behavioral or motivation or *cognitive or *psycho* or *socio* or stage* or action or goal or organization* or *ecological or (decision making)) NEAR / 0 (model* or framework* or theor*)).tw,kf. |
| TS=(((behavio$ral or motivation or cognitive or psycho* or socio* or stage* or action* or goal* or organization* or ecological or "decision making") NEAR/1 (model* or framework* or theor*))) | ((behavio$ral or motivation or cognitive or psycho* or socio* or stage* or action* or goal* or organization* or ecological or "decision making") NEAR / 1 (model* or framework* or theor*)) |
|  | ((theory NEAR / 1 (planned behavio$r) or (reasoned action)) or (stages of change) or (diffusion of innovations) or (COM-B)).tw,kf. |
| TS=((theory NEAR/1 ("planned behavio$r" or "reasoned action*")) or "stages of change" or "diffusion of innovation*" or COM-B) |  |
|  | ((behavio$ral game) or (protection motivation) or (health belief) or (social cognitive) or (locus of control) or (social learning) or (social comparison) or (cognitive adaptation) or (social identity) or (elaboration likelihood) or self-determination or attribution or (social judgment) or (dual processing) or learning or operant or self-regulation or automotive or (volitional control) or (social cognitive) or (cognitive behavior) or (transtheoretical) or (social identity) or effort-reward or demand-control or diffusion or (group minority) or (person situation contingency) or (value expectancy) or (capability opportunity motivation) or (social regulation) or (antecedent behavior consequence) or (ABC)  NEAR / 0 (model* or framework* or theor*)).tw,kf. |
| TS=("behavio$ral game*" or "protection motivation" or "health belie*" or "social cognitive" or "locus of control" or "social learning" or "social comparison" or "cognitive adaptation*" or "social identit*" or "elaboration likelihood" or "self-determination" or attribution* or "social judgment*" or "dual processing" or learning or operant or "self-regulation" or automotive or "volitional control" or "social cognitive" or "cognitive behavior" or transtheoretical or "effort-reward" or "demand-control" or diffusion or "group minorit*" or "person situation contingency" or "value expectanc*" or "capability opportunity motivation" or "social regulation*" or "antecedent behavior consequence*" or ABC) |  |
|  | ((behavio$ral NEAR / 0 economic*) or (bound* NEAR / 0 rational*) or (cognitive NEAR / 0 (effect* or bias*)) or heuristic* or fallac*).tw,kf. |
| TS=((behavio$ral NEAR/1 economic*) or (bound* NEAR/1 rational*) or (cognitive NEAR/1 (effect* or bias*)) or heuristic* or fallac*) |  |
|  | AND |
|  | (((Vector or mosquito* or insect* or tick*) NEAR / 0 (control or management)) or IVM or repellent* or insecticide or ovitrap* or (BG sentinel trap*) or (insecticide treated material*) or (bed net*) or LLIN or (long lasting insecticidal net*) or (insecticide treated net*) or (ITN) or larvicide or ((larv* or guppy) NEAR / 0 fish) or ((remov* or clear* or dispos* or eliminat*) NEAR / 1 (breed* or source*)) or (cover* NEAR / 1 (container* or lid*))) |
| TS=(((Vector or mosquito* or insect* or tick*) NEAR/1 (control or management)) or IVM or repellent* or insecticide or ovitrap* or "BG sentinel trap*" or "insecticide treated material*" or "bed net*" or LLIN or "long lasting insecticidal net*" or "insecticide treated net*" or ITN or larvicide or ((larv* or guppy) NEAR/1 fish) or ((remov* or clear* or dispos* or eliminat*) NEAR/1 (breed* or source*)) or (cover* NEAR/1 (container* or lid*))) |  |
